# Outbreaks of Granulomatous Disease Associated With *Vibrio harveyi* Infection in Grouper (*Epinephelus fuscoguttatus* × *E. lanceolatus*) Aquaculture in Taiwan

**DOI:** 10.1111/jfd.70213

**Published:** 2026-05-21

**Authors:** Shang‐Ting Wu, Chia‐Yu Hsieh

**Affiliations:** ^1^ Department of Veterinary Medicine National Chung Hsing University Taichung City Taiwan (R.O.C.)

**Keywords:** *Epinephelus*, granuloma, grouper, in situ hybridisation, *Vibrio harveyi*

## Abstract

Between 2015 and 2024, outbreaks of muscular and visceral granulomatous disease, resulting in moderate‐to‐severe mortality in farmed groupers, occurred in southern Taiwan. Initially, affected groupers were screened for granuloma‐associated pathogens, including *Mycobacterium* spp., *Nocardia* spp., *Photobacterium damselae* and *Edwardsiella* spp., using PCR methods at local animal disease prevention and control centres. Twenty‐five hybrid groupers ( *Epinephelus fuscoguttatus* × *E. lanceolatus* ) that tested negative for these pathogens were submitted to our laboratory for further examination. Infected fish exhibited multiple grey‐white nodules on the body surface and visceral organs, particularly the kidneys. Large numbers of Gram‐negative bacilli, measuring 2–3 μm in length, were observed in organ smears. Microscopic examination revealed multifocal granulomatous lesions in the subcutaneous muscle, kidneys, spleen and liver. Sequencing of the 16S rRNA gene and the *Vibrio harveyi* hemolysin (*vhh*) gene from all isolates confirmed their identity as *V. harveyi* . The pathogen was definitively localised within the lesions via in situ hybridisation using a digoxigenin (DIG)‐labelled probe. This is the first report of natural *V. harveyi* infection causing muscular and visceral granulomatous disease in Taiwanese grouper farms. These findings underscore the emerging pathological relevance of *V. harveyi* and provide a practical diagnostic framework for granulomatous diseases in aquaculture.

## 1 Introduction

Granulomatous disease in groupers is most frequently associated with bacterial pathogens belonging to several genera, including *Mycobacterium* spp., *Nocardia* spp., *Photobacterium damselae* and *Edwardsiella* spp. (Eissa et al. 2011; Liu et al. 2011; Nguyen et al. 2023; Pham et al. 2020; Shimahara et al. 2009; Ucko et al. 2016). These pathogens differ in virulence, disease progression and pathological presentation; however, all can induce systemic granulomatous inflammation that significantly compromises fish health and production performance in intensive aquaculture systems (Martínez‐Lara et al. 2021; Rajme‐Manzur et al. 2021).

In Taiwan and China, *Nocardia seriolae* is recognised as one of the principal aetiological agents of systemic granulomas in orange‐spotted groupers ( *E. coioides* ) (Nguyen et al. 2023; Shimahara et al. 2009). Infection typically manifests as multifocal, firm, creamy‐white nodules distributed in the spleen and kidneys; in severe cases, these may extend to the liver, heart and musculature (Nguyen et al. 2023; Shimahara et al. 2009). Chronic infection often results in severe organomegaly, progressive emaciation and ‘humpback’ skeletal deformities (Nguyen et al. 2023; Shimahara et al. 2009).

Similarly, studies conducted in Egypt have reported mycobacteriosis caused by *Mycobacterium marinum* in dusky grouper ( *E. marginatus* ) aquaculture systems (Eissa et al. 2011). Histologically, infections are characterised by well‐organised granulomas composed of epithelioid macrophages and multinucleated giant cells, often containing acid‐fast bacilli within the necrotic core (Eissa et al. 2011). Although disease progression is typically slow, cumulative economic losses can be substantial due to prolonged morbidity and decreased market value (Gauthier and Rhodes 2009). In contrast to the relatively slow progression of *N. seriolae* and *Mycobacterium* spp. infections.

*Photobacterium damselae* subsp. *piscicida* (Pdp) is known for causing a more acute condition historically referred to as ‘fish pseudotuberculosis’ (formerly fish pasteurellosis). This pathogen can induce the rapid formation of visceral nodules containing dense basophilic bacterial colonies (Liu et al. 2011; Romalde 2002). Outbreaks are often characterised by a sudden onset, high morbidity and acute mass mortality, posing a serious risk to juvenile fish production (Liu et al. 2011; Pham et al. 2020; Romalde 2002).

In 2015, *Edwardsiella* species were isolated from the kidneys of diseased white groupers ( *E. aeneus* ) and dusky groupers ( *E. marginatus* ) cultured in the Red Sea, Israel (Ucko et al. 2016). Affected fish presented large nodular lesions with severe suppurative nephritis (Ucko et al. 2016). While initial biochemical profiles and genetic analysis failed to provide clear species‐level identification, these isolates tentatively classified as *E. piscicida*‐like organisms, were later identified as *E. anguillarum* following taxonomic refinement of the genus (Reichley et al. 2015; Shao et al. 2015). *E. anguillarum* is now recognised as a significant granuloma‐inducing agent in various fish species, such as eels and tilapia (Armwood et al. 2019; Rahmawaty et al. 2022; Reichley et al. 2015). In contrast, the highly pathogenic *E. piscicida* has not yet been reported in groupers.

Vibriosis is a major bacterial disease affecting fish, bivalves and crustaceans, primarily caused by pathogenic *Vibrio* species including *Vibrio anguillarum* , *V. harveyi* , *V. ordalii* , *Aliivibrio salmonicida* and *V. vulnificus* (Ina‐Salwany et al. 2019; Manchanayake et al. 2023; Mohamad et al. 2019a; Xu et al. 2022). The disease has a global distribution and causes substantial economic losses in aquaculture worldwide (Ina‐Salwany et al. 2019; Mohamad et al. 2019a). Among these pathogens, *V. harveyi* is a Gram‐negative, motile and bioluminescent marine bacterium that has emerged as one of the most important pathogens in marine aquaculture (Austin and Zhang 2006; Zhang et al. 2020).

In the context of grouper farming in Taiwan, China and other Southeast Asian regions, *V. harveyi* represents a significant threat; it is primarily characterised by skin ulceration and acute septicaemia, which lead to devastating losses in both hatchery and grow‐out systems (Ina‐Salwany et al. 2019; Ito et al. 2025; Lai et al. 2023; Mohamad et al. 2019a; Zhang et al. 2020). Clinical signs in infected fish include lethargy, anorexia, erratic swimming behaviour and darkening of the skin. Gross lesions typically exhibit deep necrotic skin ulcers, severe fin rot and corneal opacity, which can progress to blindness (Ito et al. 2025; Lai et al. 2023; Mohamad et al. 2019a; Zhang et al. 2020). Internal examination frequently reveals classic septicaemic changes, such as splenomegaly, a fragile liver with focal haemorrhages and the accumulation of serosanguinous fluid in the peritoneal cavity (Ina‐Salwany et al. 2019; Zhang et al. 2020). Histologically, the disease is characterised by extensive parenchymal necrosis and vascular damage. Affected skin and muscle tissues show marked myofibrillar degeneration with bacterial and inflammatory cell infiltration, while the spleen and kidney often demonstrate depletion of haematopoietic tissue and the presence of large bacterial aggregates within the parenchyma and surrounding blood vessels (Ito et al. 2025; Lai et al. 2023; Mohamad et al. 2019b; Zhang et al. 2020).

Between 2015 and 2024, multiple outbreaks of granulomatous disease occurred in groupers cultured in southern Taiwan and Penghu County. Specimens that initially tested negative for known granuloma‐associated pathogens, including *Mycobacterium* spp., *Nocardia* spp., *Photobacterium damselae* and *Edwardsiella* spp., were collected and subjected to a comprehensive diagnostic evaluation. By integrating necropsy, bacteriological isolation, molecular identification and histopathological examinations, we documented the first report of muscular and visceral granulomas associated with natural *V. harveyi* infection in hybrid groupers ( *E. fuscoguttatus* × *E. lanceolatus* ) in Taiwan. This observation expands the known pathological spectrum of *V. harveyi* , establishing that it is not only an acute septicaemic pathogen but also a granuloma‐inducing bacterium. Furthermore, these findings underscore the importance of strengthening disease surveillance and implementing thorough differential diagnostic protocols when encountering granulomatous outbreaks in aquaculture. Further research is necessary to localise the source of infection and clarify the epidemiology, transmission and pathogenic mechanisms underlying granuloma formation in *V. harveyi* infections.

## 2 Materials and Methods

### 2.1 Samples

Between 2015 and 2024, mortality events were observed in cultured hybrid groupers ( *E. fuscoguttatus* × *E. lanceolatus* ) in southern Taiwan and Penghu County. Juveniles collected from the affected farms were submitted for analysis using cytology, bacteriology, molecular biology and histopathology (Table 1). Necropsy was performed with particular attention to the presence, distribution and characteristics of nodular lesions on the body surface and major internal organs.

**TABLE 1:** Results of necropsy, histopathology, in situ hybridisation analysis on grouper specimens investigated in this study.

Case no.	Date/Location/Water temperature	Age	Mortality (%)	Nodular lesions detected by necropsy				Granulomatous lesions observed by histopathology				Isolation of *V. harveyi* from granulomas	Signal intensity of ISH			
				M	K	S	L	M	K	S	L		M	K	S	L
AF15‐428‐1	September, 2015/Pingtung County/25°C–27°C	Juvenile (1‐month‐old)	60%	+	−	−	−	++	−	−	−	+	++	−	−	−
AF15‐428‐2				+	−	−	−	++	−	−	−	+	++	−	−	−
AF15‐428‐3				+	−	−	−	++	−	−	−	+	++	−	−	−
AF15‐480‐1	October, 2015/Pingtung County/25°C–27°C	Juvenile (1‐month‐old)	60%	+	−	−	−	+	−	−	−	+	+	−	−	−
AF15‐480‐2				+	−	−	−	+	−	−	−	+	+	−	−	−
AF15‐480‐3				+	−	−	−	+	−	−	−	+	+	−	−	−
AF15‐492‐1	November, 2015/Pingtung County/24°C–26°C	Juvenile (1‐month‐old)	60%	+	−	−	−	+	−	−	−	+	+	−	−	−
AF15‐492‐2				+	−	−	−	+	−	−	−	+	+	−	−	−
AF15‐492‐3				+	−	−	−	+	−	−	−	+	+	−	−	−
AF23‐019‐1	February, 2023/Kaohsiung City/22°C–24°C	Juvenile (3‐months‐old)	20%	−	+++	+	+	−	+++	+	−	+	−	+++	+	+
AF23‐019‐2				−	+++	+	+	−	+++	+	−	+	−	+++	+	+
AF23‐019‐3				−	+++	+	+	−	+++	+	−	+	−	+++	+	+
AF23‐220‐1	October, 2023/Kaohsiung City/23°C–25°C	Juvenile (8‐months‐old)	10%	−	+++	−	−	−	+	−	−	+	−	+	−	−
AF23‐220‐2				−	+++	−	−	−	+	−	−	+	−	+	−	−
AF23‐220‐3				−	+++	−	−	−	+	−	−	+	−	+	−	−
AF23‐272‐1	November, 2023/Pingtung County/24°C–26°C	Juvenile (4‐months‐old)	20%	−	+++	−	−	−	+++	−	−	+	−	+++	−	−
AF23‐272‐2				−	+++	−	−	−	+++	−	−	+	−	+++	−	−
AF23‐273‐1	November, 2023/Pingtung County/24°C–26°C	Juvenile (4‐months‐old)	20%	−	+++	+++	+	−	+++	+++	+	+	−	+++	++	+
AF23‐273‐2				−	+++	−	−	−	+	−	−	+	−	+	−	+
AF24‐110‐1	April, 2024/Penghu County/22°C–24°C	Juvenile (3‐months‐old)	20%	−	++	++	−	−	++	++	−	+	−	++	++	−
AF24‐110‐2				−	++	+	−	−	++	+	−	+	−	++	++	+
AF24‐110‐3				−	+	+	−	−	+	+	−	+	−	++	++	+
AF24‐329‐1	September, 2024/Pingtung County/25°C–26°C	Juvenile (1‐month‐old)	60%	+	−	−	−	+	−	−	−	+	+	−	−	−
AF24‐329‐2				+	−	−	−	+	−	−	−	+	+	−	−	−
AF24‐329‐3				+	−	−	−	+	−	−	−	+	+	−	−	−

*Note:* Necropsy and histopathology results: (+++) > 20 nodules/granulomas per organ section; (++) between 10 and 20 nodules/granulomas; (+) < 10 nodules/granulomas; (−) no granulomas. In situ hybridisation results: (+++) high‐intensity signal; (++) medium‐intensity signal; (+) low intensity; (−) no signal observed.

Abbreviations: K, kidney; L, liver; M, muscle; S, spleen.

For cytological assessment, tissue samples were obtained by needle aspiration and prepared as impression smears. The smears were subjected to Gram staining and Ziehl–Neelsen acid‐fast staining to evaluate bacterial morphology and detect acid‐fast organisms. For bacteriological analysis, duplicate tissue aspirates were inoculated onto blood agar plates (tryptic soy agar supplemented with 5% sheep blood) using the standard streak isolation method and incubated aerobically at 24°C–27°C. Bacterial growth was monitored daily, and colonies were characterised based on size, colour, morphology and haemolytic activity to distinguish primary pathogens from contaminants. Representative colonies were subsequently selected for further identification.

### 2.2 Molecular Detection and Sequencing

Tissue samples of the skin, underlying musculature, kidney, spleen and liver, along with the isolates, were aseptically collected for molecular analysis. Total genomic DNA was extracted using the LabTurbo DNA Mini Kit (LabTurbo Biotech Corporation, Taipei City, Taiwan) according to the manufacturer's instructions and served as a template for PCR assays. Highly sensitive and specific primers described in previous studies were used to screen for bacterial pathogens associated with granulomatous diseases in marine fish. These included primers targeting the *Mycobacterium* spp. heat shock protein 65 gene (*hsp65*) (Myco hsp65 441F: 5′‐ACCAACGATGGTGTGTCCAT‐3′; Myco hsp65 441R: 5′‐CTTGTCGAACCGCATACCCT‐3′) (Ringuet et al. 1999), the *Nocardia seriolae* secretory protein subunit A gene (*secA*) (Nocardia secA F: 5′‐GACAGYGAGTGGATGGGYCGSGTGCACCG‐3′; Nocardia secA R: 5′‐GCGGACGATGTAGTCCTTGTC‐3′) (Conville et al. 2006), the *Photobacterium damselae* capsular polysaccharide gene (*cps*) (Photo cps 410F: 5′‐AGGGGATCCGATTATTACTG‐3′; Photo cps 410R: 5′‐TCCCATTGAGAAGATTTGAT‐3′) (Rajan et al. 2003) and the *Edwardsiella* spp. DNA gyrase B gene (*gyrB*) (EDW gyrB F: 5′‐GCATGGAGACCTTCAGCAAT‐3′; EDW gyrB R: 5′‐GCGGAGATTTTGCTCTTCTT‐3′) (Lan et al. 2008). In cases where no amplification was obtained, universal eubacterial primers targeting the 16S rRNA gene (27F: 5′‐AGAGTTTGATCCTGGCTCAG‐3′; 1525R: 5′‐AAGGAGGTGATCCAGCC‐3′) (Bourrain et al. 1999) were used and directly sequenced for preliminary species identification. For the rapid detection of *V. harveyi* , species‐specific primer sets targeting the haemolysin gene (*vhh*) (Vh hly 454F: 5′‐TCACCTTCTTTGCAGCCGATAAC‐3′; Vh hly 454R: 5′‐CAAGGCTTCCGCGTAGTCTGATTT‐3′) (Haldar et al. 2010) were employed. The complete ORF sequence of *vhh* was subsequently amplified (VH hp. 1257F: 5′‐ATCATGAATAAAACTATTACGTTACT‐3′; VH hp. 1257R: 5′‐GAAAGGATGGTTTGACAAT‐3′) (Conejero and Hedreyda 2004) for sequencing and phylogenetic analysis. All PCR reactions were carried out in a 20 μL reaction containing 400 pM of each primer and the extracted DNA template. The thermal cycling conditions consisted of an initial denaturation step at 95°C for 7 min, followed by 35 cycles of denaturation at 95°C for 30 s, annealing at 55°C for 30 s and elongation at 72°C for 90 s. Following amplification, 4 μL of each PCR product were electrophoresed on a 1.5% agarose gel, stained with ethidium bromide and visualised under ultraviolet illumination. Positive amplification products of *vhh* were cloned into a T‐vector using a commercial TA cloning kit (YT&A; Yeastern Biotech). Sequencing was subsequently performed using a 373A automated sequencer and a BigDye Terminator cycle sequencing kit (Mission Biotech). The resulting sequences were compared with reference sequences deposited in the National Center for Biotechnology Information (NCBI) using the Basic Local Alignment Search Tool (BLAST). Phylogenetic analysis of the *vhh* ORF sequences was performed in MEGA 11 (Tamura et al. 2021) using the neighbour‐joining method with 1000 bootstrap replications (Saitou and Nei 1987).

### 2.3 Histopathology and In Situ Hybridisation

All available organs from each submitted fish were fixed in 10% neutral buffered formalin for 24 h before being transferred for histological processing. The tissues were dehydrated using increasing concentrations of ethanol, cleared with xylene and embedded in paraffin according to standard methods. Paraffin blocks were sectioned at 3 μm thickness with a microtome, mounted on glass slides and subjected to routine haematoxylin and eosin (H&E) staining. Prior to in situ hybridisation (ISH), a digoxigenin (DIG)‐labelled DNA probe targeting the *vhh* gene was synthesised using a PCR DIG Probe Synthesis Kit (Roche Molecular Biochemicals). The PCR reaction mixture comprised PCR buffer (10 mM Tris–HCl, 50 mM KCl, 2.5 mM MgCl_2_), a DIG‐11‐dUTP to dTTP ratio of 1:2 (200 μM PCR DIG Probe Synthesis Mix, Roche Diagnostics), 0.5 μM of each primer (VH hp. 1257F and VH hp. 1257R) targeting the *vhh* gene ORF and 100 pg. of plasmid template DNA in nuclease‐free distilled water. PCR amplification was performed with an initial denaturation at 95°C for 7 min, followed by 35 cycles of denaturation at 95°C for 30 s, annealing at 55°C for 30 s and extension at 72°C for 90 s. Following synthesis, the PCR product was purified using GD spin columns (Bioman Scientific Co. Ltd., Taiwan) and analysed via electrophoresis on a 1.5% agarose gel to confirm its integrity. The DNA concentration was determined using a NanoDrop spectrophotometer (Nano‐300 micro‐spectrophotometer, Medclub Scientific Co. Ltd.) and standardised to ensure a final working concentration of 200 ng/mL. Deparaffinised serial sections were cleared in xylene (2 × 10 min) and rehydrated through a graded ethanol series (100% and 70%) to distilled water. The sections were then treated with 10 mg/mL Proteinase K (Roche) in 50 mM Tris–HCl (pH 7.4) at 37°C for 15 min, followed by permeabilisation with 10 mg/mL Triton X‐100 (Sigma‐Aldrich) at 37°C for 45 min. For hybridisation, the *vhh* probe was diluted in hybridisation buffer to a final concentration of 200 ng/mL. This mixture was applied directly to the sections, which were then incubated at 42°C for 3 h. Following hybridisation, sections were treated with a 1% blocking reagent (Roche) in maleic acid buffer for 30 min to prevent non‐specific binding. Subsequently, the sections were incubated with a sheep anti‐DIG‐alkaline phosphatase (AP) conjugate (diluted 1:500 in blocking buffer) for 1 h at room temperature. To detect the signal, excess antibody was removed by washing with detection buffer (100 mM Tris–HCl, 100 mM NaCl, pH 9.5). The sections were then immersed in NBT/BCIP substrate solution (Sigma‐Aldrich) and incubated in a dark, humidified chamber until a dark blue/purple precipitate became visible (typically 30 min to 2 h). Experimental validity was maintained using two negative controls, including the *vhh* probe was applied to healthy, uninfected tissue and an unrelated *Francisella noatunensis* subsp. *orientalis* (formerly *asiatica*) probe (Hsieh et al. 2007) was applied to *V. harveyi* ‐infected sections to verify signal specificity.

## 3 Results

### 3.1 Sample Collection

In this study, a total of twenty‐five diseased groupers were collected from nine outbreak farms located in Kaohsiung City, Pingtung County and Penghu County between 2015 and 2024 (Table 1). Outbreaks were observed during the cooler months, specifically from November to April. Twelve specimens from four outbreak events in 2015 and 2024 (AF15‐428, AF15‐480, AF15‐492 and AF24‐329) exhibited marked cutaneous lesions characterised by ulceration of the lateral musculature and nodular masses in the ocular, periorbital and opercular tissues, with cumulative mortality rates reaching 60% (Table 1, Figure 1a). In contrast, the remaining thirteen specimens collected from five outbreak events between 2023 and 2024 (AF23‐019, AF23‐220, AF23‐272, AF23‐273 and AF23‐110) showed no external wounds or skin lesions. Instead, the most prominent gross lesions were found in the internal organs; these included organ enlargement, rounding and multiple white nodules of varying sizes in the kidneys, spleen and liver (Table 1; Figure 1b). Notably, no specimens presented both cutaneous and visceral lesions. Cytological examination of smears prepared from both cutaneous and visceral lesions revealed numerous Gram‐negative, non‐acid‐fast bacilli measuring 2–3 μm in length. The bacteria were localised either extracellularly or within host cells. In all cases, bacteriological culture consistently yielded pure colonies that appeared grey‐white to yellowish in colour and exhibited α‐haemolysis on blood agar. These colonies grew rapidly within 24 h of aerobic incubation at 25°C. The morphology of the cultured isolates corresponded to the Gram‐negative, non‐acid‐fast rods observed in tissue smears.

**FIGURE 1:**
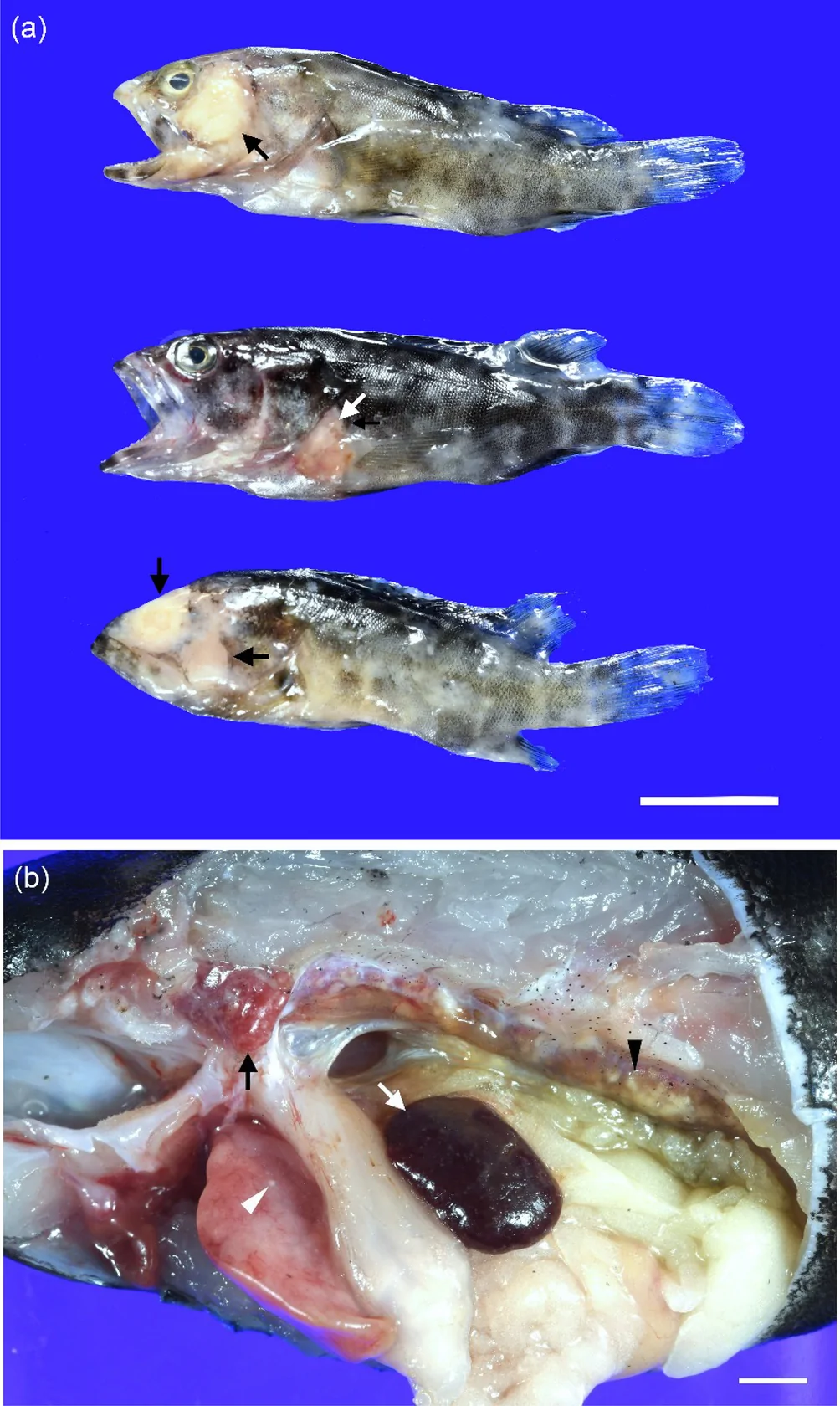
External lesions and necropsy findings in diseased hybrid groupers collected between 2015 and 2024. (a) Cutaneous‐muscular lesions (AF24‐329) characterised by ulceration of the lateral musculature (white arrow) and nodular masses in the ocular, periorbital and opercular tissues (black arrows). (b) Visceral lesions (AF23‐273‐1) showing multiple white nodules of varying sizes in the enlarged anterior kidney (black arrow), posterior kidney (black arrowhead), spleen (white arrow) and liver (white arrowhead). Scale bar = 1 cm.

### 3.2 PCR Detection and Phylogenetic Analysis

All grouper specimens collected in this study were initially subjected to routine cytological and bacteriological examination at the local Animal Disease Prevention and Control Centre. However, no isolates consistent with commonly recognised granuloma‐associated pathogens (*Mycobacterium* spp., *Nocardia* spp., *P. damselae* and *Edwardsiella* spp.) were recovered. Furthermore, our PCR assays, using the well‐established species‐specific primer sets mentioned previously, yielded negative amplification results in all submitted cases, thereby excluding these bacteria as potential aetiological agents. In contrast, PCR detection targeting the *V. harveyi* haemolysin gene (*vhh*) consistently produced 454 and 1257 bp amplicons from our samples collected from either cutaneous or visceral lesions (Figure 2a,b). These findings were consistent with the bacteriological results, where pure cultures of *V. harveyi* were isolated from affected tissues (Table 1). Comparative sequence analysis of the *vhh* ORF sequence (1257 bp) demonstrated that all isolates obtained in this study, regardless of tissue origin, clustered with pathogenic *V. harveyi* strains isolated from other grouper species, showing 99.5%–99.8% sequence similarity (Figure 2c). While the combination of bacteriology and molecular biology provided evidence of *V. harveyi* 's presence, its specific role in granuloma formation required further histological confirmation.

**FIGURE 2:**
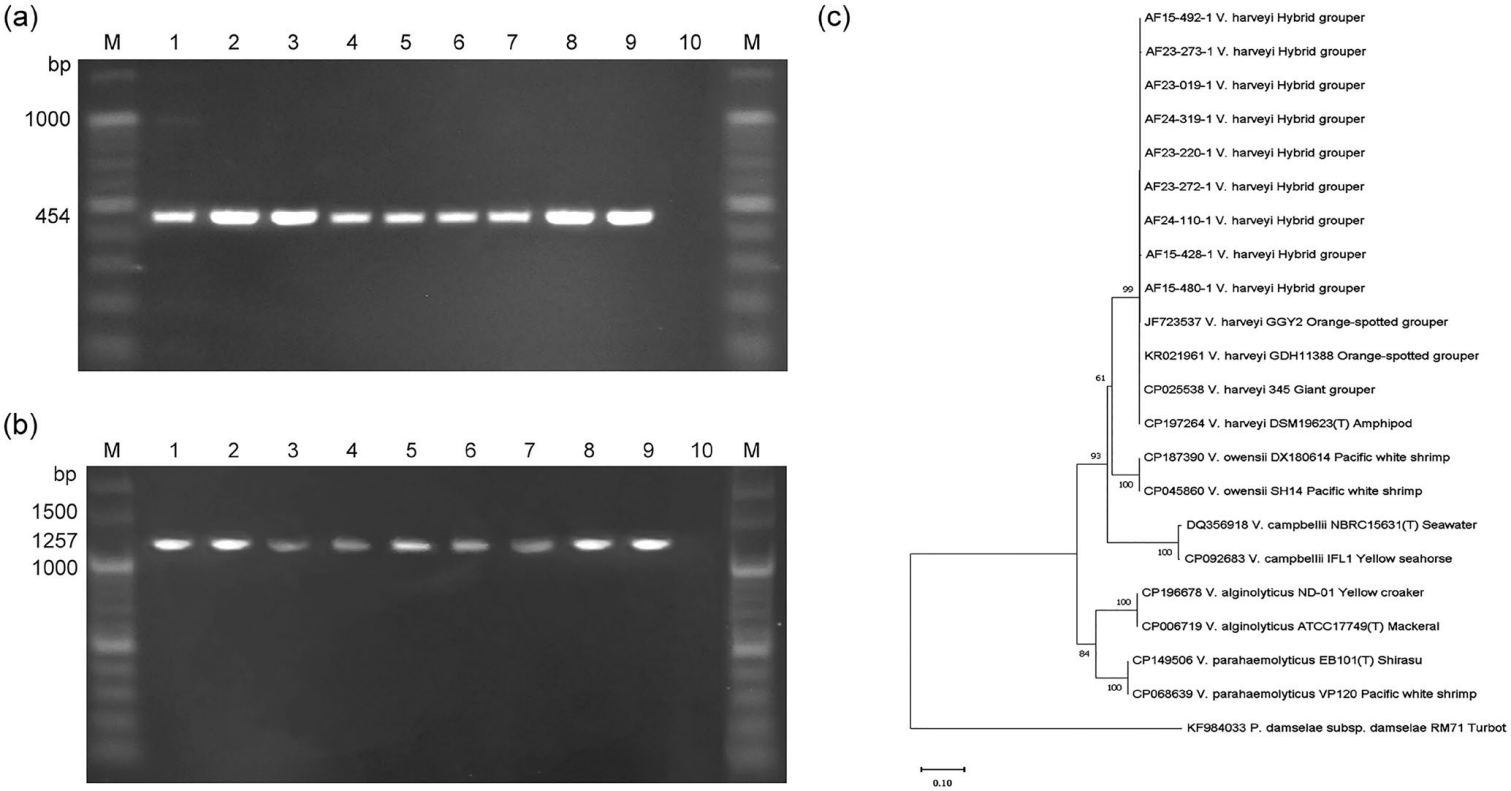
Agarose gel electrophoresis stained with ethidium bromide showing PCR amplification results using *V. harveyi* ‐specific primers and genomic DNA from nine aquaculture farms as templates. (a) Specific 454 bp and (b) 1257 bp fragments of the *V. harveyi* haemolysin (*vhh*) gene were successfully amplified in submitted cases, respectively. Lane M: 100 bp ladder marker; Lanes 1–9: Aquaculture farms AF15‐428, AF15‐480, AF15‐492, AF23‐019, AF23‐220, AF23‐272, AF23‐273, AF24‐110 and AF24‐329; Lane 10: Negative control. (c) Phylogenetic tree based on nucleotide sequence alignments of the ORF region of the *V. harveyi vhh* gene. Sequences were aligned using the ClustalW method, and phylogenetic reconstruction was performed using the neighbour‐joining method with 1000 bootstrap replicates in MEGA 11. *Photobacterium damselae* subspecies *damselae* was selected as the outgroup.

### 3.3 Histopathology and In Situ Hybridisation

In cases of cutaneous‐muscular vibriosis, histopathological examination revealed extensive bacterial invasion of the dermis, subcutaneous tissue and underlying musculature. This was accompanied by coagulative necrosis and dense heterophilic infiltration forming multiple suppurative foci (Figure 3a). As lesions progressed, these foci became encapsulated by macrophages and epithelioid cells, eventually developing into well‐organised granulomas with central necrosis, dense basophilic bacterial aggregates and concentric layers of epithelioid cells and heterophils (Figure 3a). The surrounding tissue also showed marked exudation and inflammatory infiltration. In grouper cases presenting with visceral nodules, histopathological examination revealed multifocal granulomas, often accompanied by varying degrees of inflammation, haemorrhage and necrosis, resulting in the disruption of the organ architecture (Figure 4a,c,e,g). Granulomatous lesions were predominantly observed in the kidney, followed by the spleen and liver, which was consistent with the gross findings (Table 1). The structure of the granulomas ranged from early‐stage lesions, consisting of localised suppurative foci surrounded by a thin layer of macrophages and epithelioid cells, to well‐developed granulomas exhibiting central necrosis with cellular debris infiltrated with bacteria, surrounded by epithelioid cells and an outer layer of fibrous connective tissue. To further investigate pathogen localisation, in situ hybridisation (ISH) using a *V. harveyi* hemolycin gene (*vhh*)‐specific DNA probe was performed. The DIG‐labelled probe was synthesised via PCR as detailed in the Materials and Methods. Agarose gel electrophoresis confirmed that the length of the PCR product corresponded to the predicted size (Figure S1). As expected, the DIG‐labelled probe exhibited a higher molecular weight than its unlabelled PCR counterpart, as evidenced by its slightly slower migration during electrophoresis. Positive ISH signals were observed either dispersed throughout areas of degenerated parenchyma or localised within the necrotic areas of granulomas in the affected musculature (Figure 3b) and internal organs (Figure 4b,d,f,h). No positive signals were observed in the nodular lesions when the *Francisella noatunensis* subsp. *orientalis* probe was employed as a negative control, neither in the subcutaneous muscle (Figure 5a) nor within the internal organs (Figure 5b). Similarly, no positive signals were detected in the healthy grouper control sections (Figure 5a,b), further confirming the specificity of the hybridisation. These ISH findings confirmed a strong association between *V. harveyi* infection and the development of granulomas in groupers (*Epinephelus* spp.).

**FIGURE 3:**
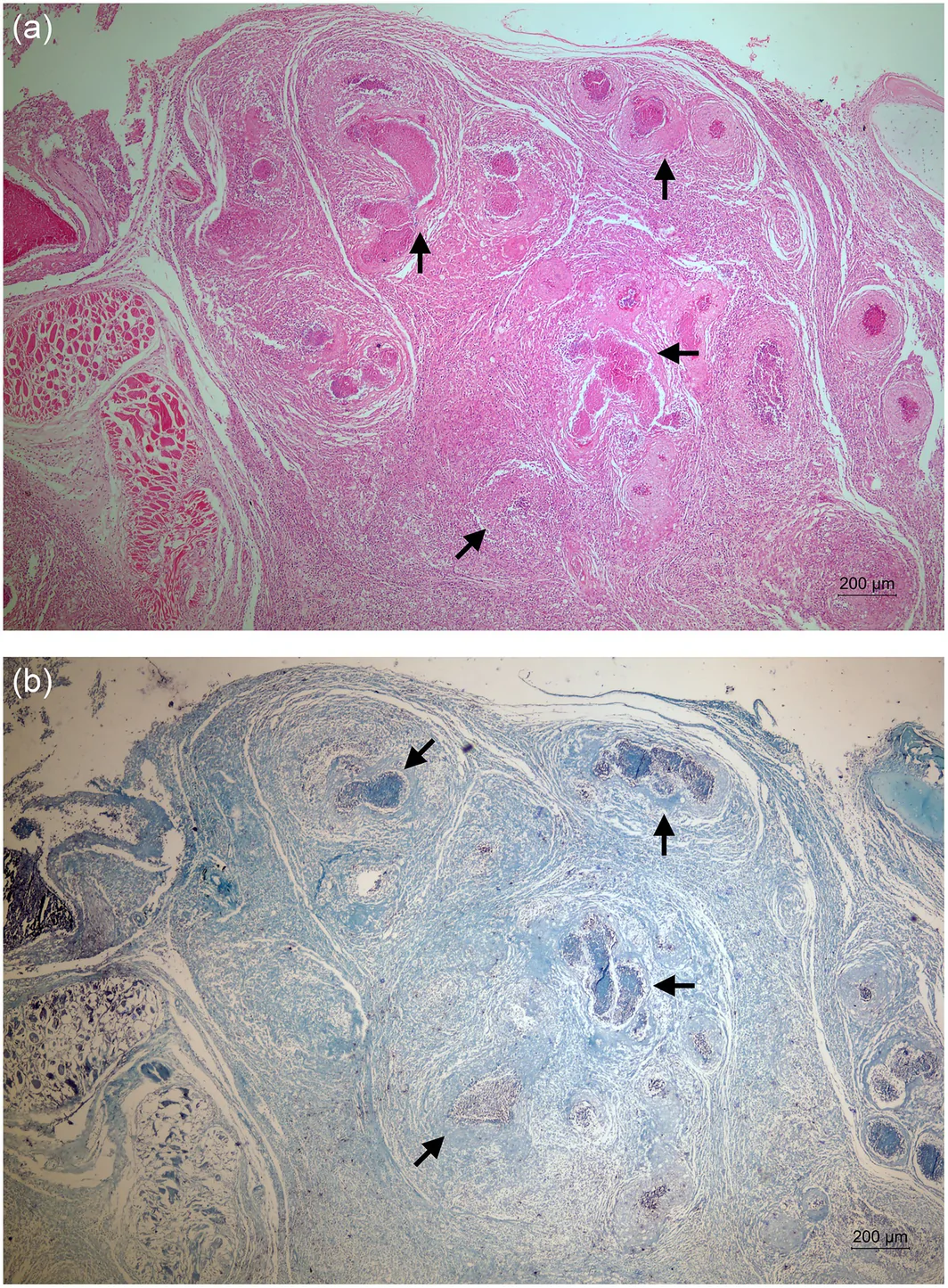
Histopathology and in situ hybridisation results from serial sections of cutaneous‐muscular lesions in infected grouper (AF24‐329‐1), stained with haematoxylin and eosin (H&E) and subjected to in situ hybridisation assay. (a) Dense heterophilic infiltration of the dermis and underlying musculature, accompanied by necrosis, suppurative foci and granulomas (black arrows). (b) Positive ISH signals dispersed throughout areas of necrosis and granulomas in the subcutaneous musculature (black arrows). Scale bar = 200 μm.

**FIGURE 4:**
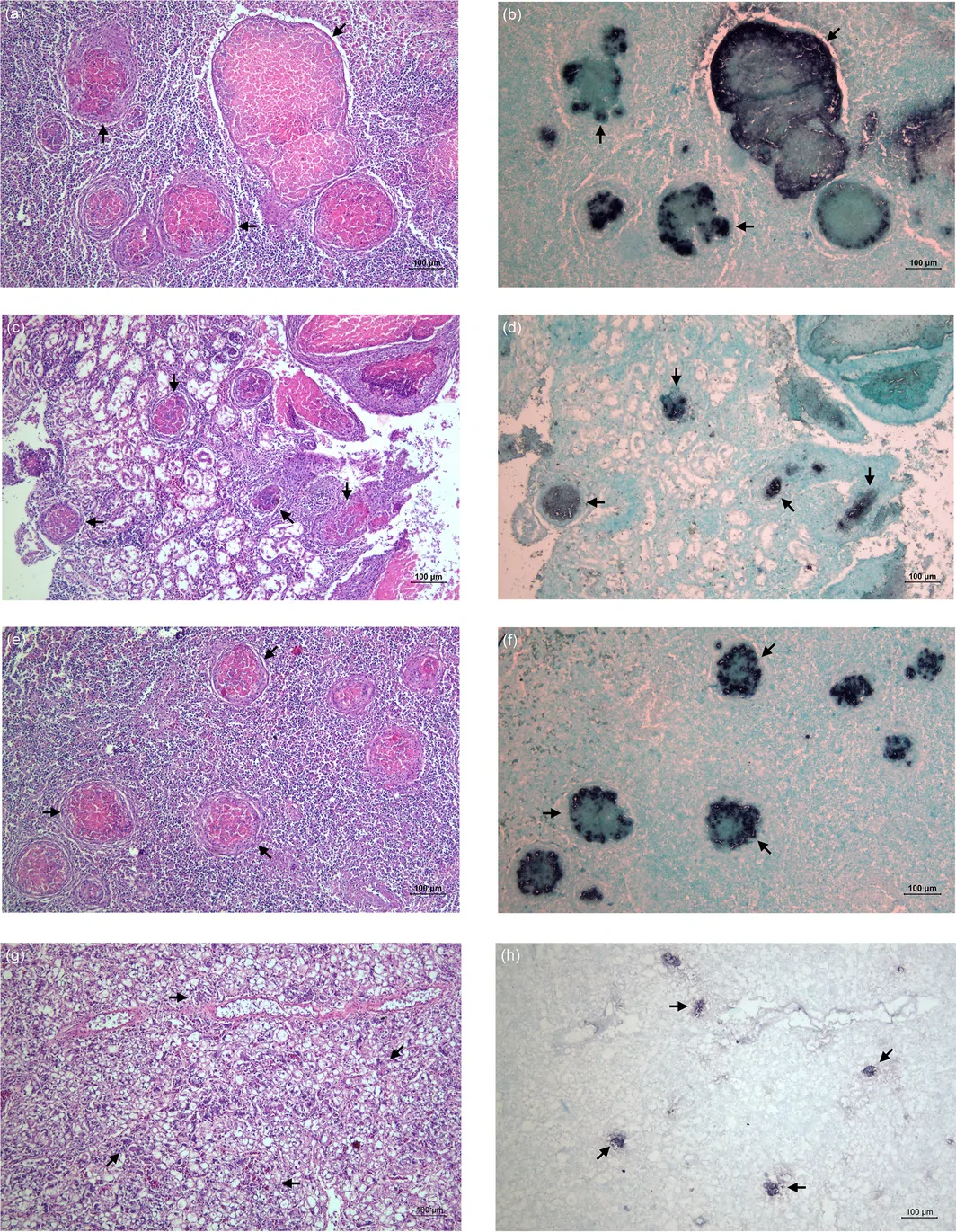
Histopathology and in situ hybridisation results from serial sections of internal organs in infected grouper (AF23‐273‐1), stained with haematoxylin and eosin (H&E) and subjected to in situ hybridisation assay. (a) Anterior kidney showing marked granulomatous foci characterised by inflammatory cells surrounding necrotic tissues with bacterial aggregates (black arrows). (b) Strong ISH signals localised within the necrotic centres of the granulomas (black arrows). (c) Posterior kidney showing renal tubular necrosis with multifocal to diffuse granulomatous lesions (black arrows). (d) Moderate ISH signals detected within the necrotic regions of the granulomas (black arrows). (e) Spleen with multifocal granulomas containing central necrosis and bacterial aggregates (black arrows), surrounded by inflammatory cells and fibrous connective tissue. (f) Strong ISH signals concentrated around the necrotic zones of the granulomas (black arrows). (g) Liver showing focal necrosis without apparent granulomas or bacteria. (h) Sparse ISH signals observed within the degenerated hepatic tissue (black arrows). Scale bar = 100 μm.

**FIGURE 5:**
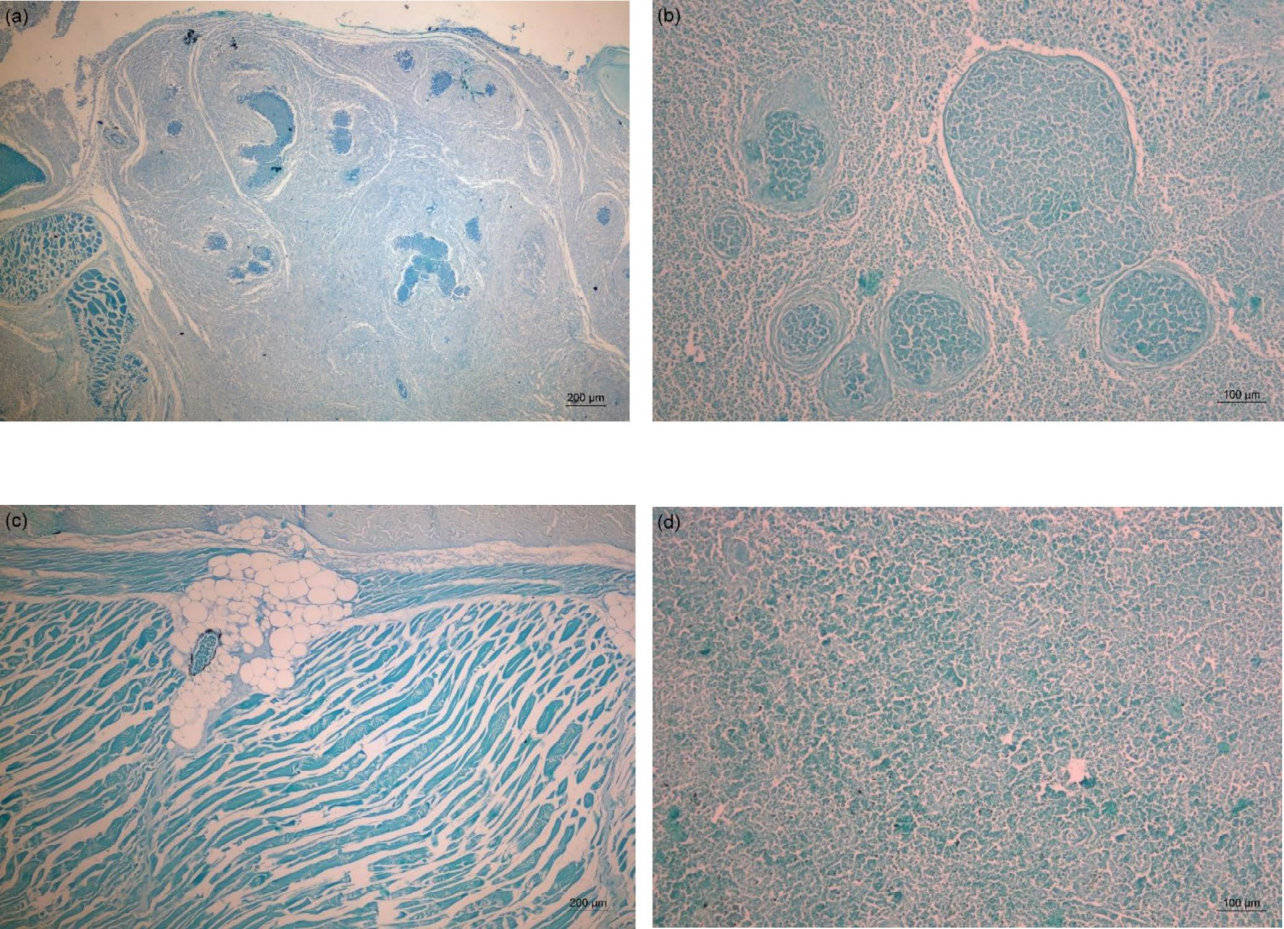
Negative control examinations, using both the *vhh* probe and an unrelated probe, were performed to verify signal specificity. No positive signals were observed in the nodular lesions when the *Francisella noatunensis* subsp. *orientalis* probe was employed as a negative control, neither in the subcutaneous muscle (a) nor within the internal organs (b). Similarly, no positive signals were detected in the muscle (c) or kidney (d) of healthy grouper control sections, further confirming the specificity of the hybridisation.

## 4 Discussion

Granulomatous lesions caused by *V. harveyi* are uncommon, yet they have been documented in previous literature. In farmed tiger puffers ( *Takifugu rubripes* ) in Japan, Mohi et al. (2010) described nodular lesions in the branchial chamber of the gills following natural infection with *V. harveyi* . Furthermore, experimental intraperitoneal injection of the bacterium induced extensive internal organ damage and chronic granulomatous lesions in visceral organs distant from the injection site as early as five days post‐inoculation (Mohi et al. 2010). More recently, an incidental observation was reported in an experimental study conducted in China, which investigated variations in gut–liver immune responses and gut microbiota following a *V. harveyi* challenge in Pearl Gentian groupers ( *Epinephelus lanceolatus* × *E. fuscoguttatus* ). In that study, nodules appeared in the liver 12 h post‐inoculation with a highly virulent strain of *V. harveyi* at a concentration of 3.37 × 10^5^ CFU g^−1^ (Deng et al. 2020). However, no other reported cases with similar pathological findings have been analysed or discussed.

The present study aimed to isolate *V. harveyi* from granulomas in diseased groupers and to confirm its role as the aetiological agent responsible for the observed granulomatous disease outbreaks. Following negative results for known granuloma‐associated bacteria, pathogen identification was first achieved by 16S rRNA gene sequencing of nodular tissues and isolates from cutaneous and visceral lesions, which consistently identified the organism as *V. harveyi* . Following this discovery, additional granulomatous cases were collected and screened specifically for *V. harveyi* (Table 1). Over the course of a decade, cases exhibiting coinfection with other recognised granuloma‐associated bacteria, including *Mycobacterium* spp., *N. seriolae* , *P. damselae* and *Edwardsiella* spp., were systematically excluded from the analysis using bacteriological and molecular diagnostic methods. Consequently, we selected twenty‐five grouper specimens originating from nine farms in which *V. harveyi* was the sole detected bacterial pathogen (Table 1). This strict exclusion criterion enabled a clearer and more accurate assessment of lesions and mortality attributable solely to *V. harveyi* . Furthermore, the integration of ISH localisation provided strong evidence that *V. harveyi* was the causative agent present in both the cutaneous‐muscular tissues and visceral organs and that the infection was closely associated with the development of the granulomatous lesions observed in this study. Ultimately, these findings suggest that systemic granulomas associated with *V. harveyi* represent an emerging and widespread outbreak pattern in grouper aquaculture systems across Taiwan.

Diagnosing bacterial granulomas presents a considerable challenge in aquatic veterinary medicine. A primary reason is that the gross features of nodules in internal organs often exhibit significant morphological overlap across different bacterial pathogens. Consequently, relying solely on clinical presentation and macroscopic examination is insufficient for accurate aetiological determination. Although culture remains the gold standard for diagnosing bacterial diseases, fastidious and slow‐growing organisms, such as *Mycobacterium* and *Nocardia* species, may take several weeks to grow. This delay in definitive diagnosis can postpone critical therapeutic interventions (Austin 2019; Gauthier and Rhodes 2009; Martínez‐Lara et al. 2021). Furthermore, prior antimicrobial usage or low bacterial loads within encapsulated lesions may further reduce culture sensitivity. For these reasons, effective differential diagnosis requires a comprehensive, multimodal approach. This includes cytological and histopathological examination supported by multiple staining techniques, bacterial isolation and molecular diagnostic methods targeting clinically prevalent pathogens. Such complexity makes the study of granulomatous diseases both challenging and essential for aquatic veterinarians and researchers.

The epidemiology of *V. harveyi* is characterised by its role as a ubiquitous and opportunistic marine bacterium that thrives under intensive aquaculture, particularly in tropical and subtropical regions such as Southeast Asia, China and Taiwan (Austin and Zhang 2006; Ina‐Salwany et al. 2019; Mohamad et al. 2019b; Zhang et al. 2020). This pathogen can persist for extended periods in the water column, sediments and copepods, which serve as environmental reservoirs for horizontal transmission via contaminated water, ingestion of infected prey or entry through skin abrasions (Austin and Zhang 2006; Zhang et al. 2020).

The economic impact is most severe in hatcheries, where the bacterium causes acute septicaemia and mass mortality in larval or juvenile stages. In adult fish, infections frequently manifest as chronic skin ulcerations and ocular lesions, leading to growth retardation and reduced market value (Xu et al. 2022; Zhang et al. 2020). Vibriosis outbreaks are strongly stress‐mediated and typically peak during the warmer months. However, while 26°C–32°C represents the optimal thermal range for *V. harveyi* replication and virulence factor expression (Natrah et al. 2011; Xu et al. 2022; Zhang et al. 2020), our findings demonstrate that the pathogen induces severe granulomatous disease at moderate temperatures (22°C–27°C) from September to April (Table 1). This indicates that Taiwanese grouper aquaculture faces a persistent, year‐round risk of *V. harveyi* infection; therefore, the possibility of *V. harveyi* involvement should not be readily excluded in any season when investigating disease outbreaks in Taiwan.

During our disease investigation, we observed differences in mortality between groupers with cutaneous‐muscular lesions collected in 2015 and 2024 and those with visceral nodules collected between 2023 and 2024 (Table 1). This variation likely reflects a combination of host age and differences in disease progression. One‐month‐old juvenile groupers typically have an underdeveloped immune system. Under these conditions, *V. harveyi* rapidly causes damage to the skin and musculature (Figure 1a). Severe necrotising lesions compromise epithelial integrity, leading to osmotic imbalance and electrolyte disturbances. When these physiological disruptions are coupled with the high metabolic demands of early growth, they frequently result in much higher mortality rates (Mohamad et al. 2019a; Zhang et al. 2020). In contrast, groupers older than three months have a more mature immune system capable of mounting a chronic cellular response. This allows the bacteria to be contained within visceral granulomas (Figure 1b). While these lesions indicate systemic infection, they also reflect partial host control that can delay the onset of fatal septicaemia (Rajme‐Manzur et al. 2021). Consequently, the lower mortality observed in older fish with visceral lesions likely represents a shift from acute death in young juveniles to a more prolonged disease course in older fish (Table 1). Nevertheless, the comparative epidemiology and pathogenesis of *V. harveyi* infections warrant further investigation.

ISH successfully achieved the study's objectives and has proven to be an ethical and powerful approach for investigating the pathogenesis and virulence of this disease (Haase 1986; Hsieh et al. 2007; Murgia et al. 2024). By using formalin‐fixed tissue sections collected during the outbreak investigation and applying pathogen‐specific probes, ISH enabled precise visualisation of the spatial distribution and severity of the pathogenic bacterium within affected tissues (Hsieh et al. 2007). Moreover, in cases involving potential coinfections, a comparison of hybridisation signal intensity can provide a relative indication of pathogen abundance within the same tissue section. This offers a more intuitive interpretation that is less influenced by environmental variables or post‐sampling changes. Specific hybridisation signals confirmed the direct association between *V. harveyi* and granulomatous lesions in this study. To ensure the highest level of diagnostic confidence, we integrated two distinct negative control groups into the ISH analysis to rigorously validate both assay sensitivity and specificity. These controls demonstrate the high sensitivity and specificity of this method for detecting target nucleic acids in tissue sections under light microscopy. The DIG‐labelled probe was synthesised using a 1:4 dUTP:dTTP ratio, adhering to established protocols for optimal labelling efficiency (An et al. 1992). The *vhh* gene was selected as the probe target due to its high specificity for *V. harveyi* and its superior discriminatory power. It shares only 88% sequence similarity with its closest relative, *V. owensii* , a common marine *Vibrio* species primarily affecting shrimp and spiny lobsters (Figure 2). In contrast, full‐length 16S rRNA sequences typically exhibit over 98% similarity among *Vibrio* species, including pathogens such as *V. alginolyticus* , *V. campbellii* and *V. owensii* . This significant divergence in the *vhh* gene sequence reinforces the specificity and reliability of the probe utilised in this study, ensuring accurate identification of *V. harveyi* .

In three particular cases (AF23‐273‐2, AF24‐110‐2 and AF24‐110‐3), neither grossly visible nodular lesions nor corresponding histopathological abnormalities were identified in the liver under routine H&E staining (Table 1). However, positive PCR results, successful pathogen isolation and direct ISH localisation confirmed the presence of *V. harveyi* within the hepatic tissues (Table 1; Figure 2). In these instances, the bacteria were observed as early‐stage aggregates rather than fully developed granulomas (Figure 4g,h). This finding reaffirms the value of PCR and ISH as highly sensitive and specific screening tools for detecting early or subclinical infections and highlights the importance of employing multimodal diagnostic approaches when investigating granulomatous diseases in groupers.

Based on the combined macroscopic, microbiological, molecular, histopathological and ISH findings, outbreaks of granulomas in grouper aquaculture in Taiwan were definitively confirmed to be *V. harveyi* infections. There is a critical need for the fisheries industry and regulatory authorities to improve monitoring of granulomatous disease outbreaks. Following this identification, routine screening for *V. harveyi* should be incorporated into the diagnostic workflow for granulomatous disease in groupers.

## 5 Conclusions

This study provides the first report of natural *V. harveyi* infection causing cutaneous and visceral granulomas in Taiwanese farmed groupers. Regulatory authorities and the aquaculture sector must enhance surveillance to detect outbreaks of *V. harveyi* granulomatous disease. Furthermore, the epidemiology and pathogenesis of *V. harveyi* warrant continued investigation.

## Author Contributions

**Shang‐Ting Wu:** data curation, formal analysis, methodology, investigation, writing – original draft, software. **Chia‐Yu Hsieh:** writing – review and editing, methodology, visualization, formal analysis, funding acquisition, investigation, supervision, resources, project administration.

## Funding

The Ministry of Agriculture of Taiwan, R.O.C., generously supported this study (Grants 112AS‐6.1.1‐FA‐F1 and 113AS‐6.2.5‐FA‐F1).

## Conflicts of Interest

The authors declare no conflicts of interest.

## Supporting information


**Figure S1:** Agarose gel electrophoresis of DIG‐labelled probe targeting the *vhh* gene. The DIG‐labelled product (Lane 1) showed slower migration compared to the unlabelled PCR product (Lane 2), consistent with successful labelling. Lane M: 100 bp ladder marker.

## Data Availability

The data that support the findings of this study are available on request from the corresponding author. The data are not publicly available due to privacy or ethical restrictions.
